# Supplementary material for: Saccadic suppression enhances saliency of ecologically relevant stimuli in the zebrafish optic tectum
Source: PLoS Biol. 2026 Nov 4;24(11):e3004093. doi: 10.1371/journal.pbio.3004093 (PMC13766609; doi:10.1371/journal.pbio.3004093)
Supplement: Supplementary file 1 [file pbio.3004093.s011.pdf]

Responses to Reviewer #3

Reviewer #3: Comparing RGC and tectal activity:

- To better compare the retinal and tectal data, the authors should do so in the same domain, rates (deconv the fluorescence), or fluorescence (convolve the rates). This is particularly problematic in Figure 8, where suppression is being compared across conditions.

We followed the reviewer's suggestion and deconvolved the tectal calcium indicator fluorescence data from Figure 3 in order to obtain relative firing rate readings. In our new Figure S5A we show that suppression dynamics match between non-deconvolved fluorescence data and deconvolved fluorescence data (same data set). Since deconvolution of fluorescence data is not commonly done in the field and can have drawbacks (see Benisty et al., 2022 & Shen et al., 2022 for detailed discussion), and after having established that there is no difference in the observed saccadic suppression when using fluorescence data versus deconvolved fluorescence data, we decided that we would like to keep our presentation of non-deconvolved data in the manuscript as originally submitted. Nonetheless, we did add the supplementary Figure S5A and a corresponding methods description, so that interested readers can inform themselves about the (in this case: only subtle) effect differences.

- Only ~2% of the tectal cell in Figure 3 respond to flashes, while almost all of the RGC cells did. This begs the question if this tectal group is the most relevant population? What evidence is there that the suppression in these cells is behaviorally relevant, as opposed to suppression in another group?

As we mentioned in our manuscript (please see lines 94ff), we did see significant suppression of O-bend behavior (Figure 1) using the exact same global dark flashes that elicited suppressed responses in our tectal cells. Thus, the tectal suppression that we saw was indeed behaviorally relevant.

A percentage of ~2% is not surprising, given that the tectum has visual neurons with very diverse receptive fields and any visual stimulus will only recruit a fraction of the neurons in the tectum. However, this does not mean that the responding fraction is not functionally relevant. For example, most of the tectal cells are responsive to small visual stimuli, i.e. they only fire when the stimulus is present in the small excitatory receptive field at a particular position in visual space, and they are suppressed by larger visual stimuli (size-selective, likely due to surround inhibition, 80% of the small-size receptive field neurons are size-selective according to Wang et al., 2020). Thus, our global flashes were not optimal for these neurons. Furthermore, we stimulated with reddish light and recent evidence suggests that a much higher responsivity is present when stimuli are naturalistically "white", i.e. include UV light (Fornetto et al., 2025). All of these factors mean that many studies on visual function of the optic tectum only include a fraction of the total cell count in the optic tectum. Having said that, we clearly observed robust responses to our global flashes, and these global flashes were enough to alter behavior. Thus, our observations are still functionally relevant.

Concerning the numerical value of our observed fraction of neurons, our stimulus size meant that it was most likely for neurons with large receptive fields to respond to it. In a different publication, Förster et al. (Förster et al., 2020) (c.f. their Figure 2C and 2D, copied here for your convenience) only included 1759 tectal cells (3 animals) and found dark flash responses in a fraction of the included

neurons (~15% in Figure 2B, 1.8 % exclusively dark-flash-responding neurons in Figure 2D of that publication). Thus, our observed fraction is consistent with prior knowledge.

To summarize, there are multiple factors that lead to the low neuron count for our stimulus. However, suppression of global dark flash responses is still behaviorally relevant, since we saw significant suppression of O-bend behavior (Figure 1), which is elicited by a global dark flash and likely processed by those exact tectal neurons.

<Figure 2 of Foerster et al. 2020>

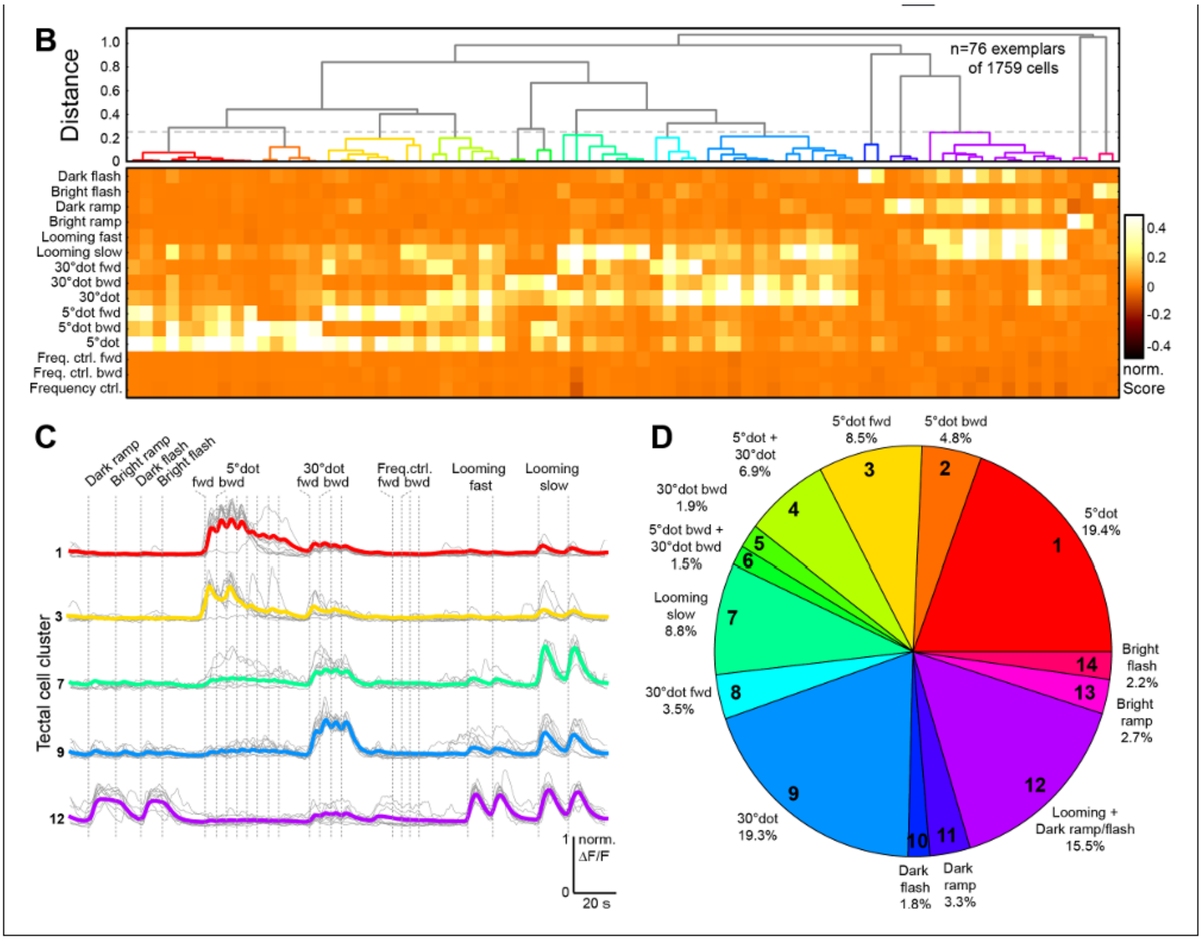

Figure 2. Behaviorally relevant response clusters in the tectum. (A) Analysis of calcium imaging data. Within selected response windows (black rectangles), the ΔF/F traces were correlated to the corresponding regressor and 15 score values were calculated for each cell (CR: coefficient of regression, R^2: correlation, response: black trace, model: green trace). (B) Hierarchical clustering of functional cell types in the tectum. Normalized scores for 76 exemplars, obtained by affinity propagation of 1759 cells (of three larvae) in total are shown. Dashed line indicates a chosen distance threshold of 0.25, which results in 14 functional clusters. (C) Normalized calcium transients of all exemplars (gray) and average traces of all cells (colored) for the five largest clusters. (D) Functional cluster distribution. Tectal cluster numbers are indicated.
The online version of this article includes the following figure supplement(s) for figure 2:

Characterization of the suppressive effect in the tectum:

- The authors have not properly dealt with the issue that the suppressive effect in the tectum is present ahead of the time there is any texture displacement (Figure 5D). Indeed, the simplest

interpretation of this figure is that the texture displacement at time 0 is not having any effect as the suppression at time -500 looks to be indistinguishable from suppression over the next 4 seconds.

The seemingly paradoxical observation that the suppressive effect is present before the texture displacement is in fact not paradoxical at all and can be explained by the long processing time (from retina to tectum) and the temporal profiles of calcium responses. A detailed explanation is provided further below (particularly, in our response to the next reviewer comment).

The reviewer raises the point that the figure could be interpreted differently (no suppression exists). We respectfully disagree: when comparing peri-saccadic responses to baseline responses, in which the flash stimulus was shown in temporal isolation from texture displacement, it can be seen clearly that an effect of the texture displacement on the flash responses is present (and significant). Our new Figure S5B provides further support for this finding: the suppressive effect disappears by 8 seconds after texture displacement onset. Responses to flashes shown 8, 16, and 32 seconds after texture displacement onset are indistinguishable from baseline responses. So, while “peri-saccadic” flash responses from -500ms to 4000ms relative to texture displacement onset are all similar in size, they significantly differ from flash responses for the 3 later time points and from the baseline flash responses. This finding can only be associated to the presence of a texture displacement.

What could be going on? The authors suggest there could be stimulus-stimulus interactions. The authors have the stimulus times recorded, so they should be able to demonstrate if interactions explain the issue.

What is going on here is a phenomenon termed visual backward masking (now mentioned in line 275). Visual masking occurs when the visibility of one image, called a target (here: flash), is reduced by the presence of another image, called a mask (here: texture displacement). Masking means that the target elicits reduced responses (compared to trials without mask). In backward masking, the mask (texture displacement) is shown AFTER the target (flash), and the responses to the target (flash) are reduced nonetheless. Visual masking has been described in mammalian vision research in great depth, including some of our own work (Idrees et al., 2020, 2022; Rolls et al., 1999; Sperling, 1965). In Idrees et al. 2020 and 2022, we showed pre-saccadic suppression (i.e. backward masking) in isolated mouse, pig and primate retina in complete absence of any motor signals. Further, in these same studies we showed that human vision shows pre-saccadic suppression in both real and simulated saccades. This backward masking effect is not exclusive to texture displacements but can also be elicited by a simple luminance step (see Figure 8 in Idrees et al., 2022, copied here below). Here too, pre- and post-saccadic suppression are approximately of the same strength, but they are both contingent on the sensory transient (luminance step in this case, and texture displacement in our case). It is therefore very likely that pre-saccadic suppression observed in zebrafish is also due to visual backward masking, as described in other species. [There is no consensus in the literature yet as to the origins of backward masking, and this is beyond the scope of our study. Possibilities e.g. include a) slower arrival of flash responses than texture displacement responses in the tectum, b) a role of the cessation of the flash, rather than its onset, c) slow feedback circuits from other brain areas to the optic tectum. Having said that, our results could be considered as revealing a neural correlate of backward masking in zebrafish, which we find very exciting.]

<Figure 8 from Idrees et al. 2022>

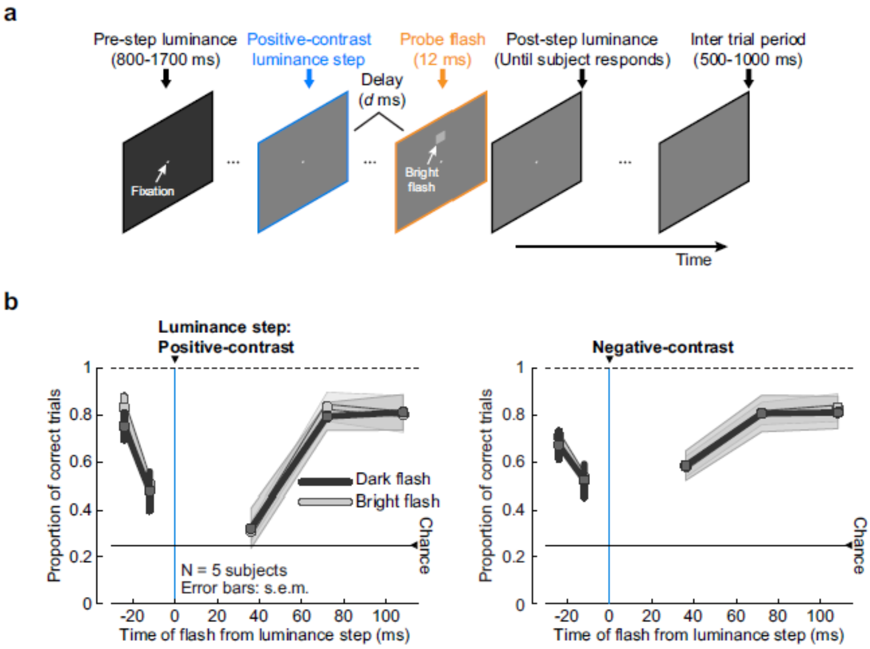

Fig. 8 Perceptual suppression following luminance steps. a Example visual task trial. Subjects fixated a small spot on a uniform background with a set luminance (pre-step luminance) for a random duration (800–1700 ms). Background luminance then increased (like shown here) or decreased (positive or negative-contrast luminance step, respectively). At one of five times relative to the luminance step (−24, −12, 36, 72, or 108 ms), a luminance pedestal (probe flash, 147.8 × 147.8 min arc) was applied for ~12 ms at one of four locations relative to the fixation spot: 7 deg above (shown here), below, to the right, or to the left. The probe flash was brighter (shown here) or darker than the current screen luminance. The background remained at the post-step luminance until the subject responded with the perceived location of the flash, plus an additional 500–1000 ms but without the fixation spot, allowing the subject to relax. The current luminance was the pre-step luminance of the consecutive trial. **b** Performance of human subjects (mean ± s.e.m., $N = 5$ subjects), to correctly localize a dark (dark gray) or a bright (light gray) probe flash presented at different times relative to positive-contrast (left panel) and negative-contrast (right panel) luminance steps (blue line). Each subject's responses were averaged across the different positive-contrast (0.3–0.56 Michelson contrast) and negative-contrast (−0.3 to −0.56 Michelson contrast) luminance steps. Perceptual performance was reduced around the time of luminance steps, reflecting suppression, irrespective of the combination of luminance-step polarity and flash polarity. There were no statistically significant differences in suppression of dark and bright probe flashes (two-tailed Wilcoxon rank-sum test). Note that in the right panel, the suppression profile for bright probe flashes almost completely overlaps the suppression profile of dark probe flashes.

The reviewer also states that we should be able to demonstrate if stimulus-stimulus interactions explain the issue. We think that the presence of the suppression curve (Fig. 3D) directly shows that an interaction between flash and texture displacement responses indeed exists. We furthermore looked at the interaction of two flash stimuli (either 500 ms and 500ms with a predefined delay, or 20 ms for the first and 500 ms for the second flash) in our new Figure S5D. We again find that this interaction exists as well, but it is much weaker (only < 5% suppression). Thus, pre-saccadic suppression is real and can be explained by backward masking.

Another possibility, given the results of Figure 6F, is that there are motor efference signals that are providing suppression. As seen in this figure, even in the absence of texture there is suppression that may be coupled to the time of saccade onset - hard to be definitive here since no pre-saccadic suppression is shown. If we allow that there is saccade-related suppression in the absence of texture due to efference from oculomotor control centers, then we have to expect that this would be the case even when the eyes are restrained in agarose. Thus, the pre-texture displacement suppression seen in Figure 5D could largely be due to the presence of a saccadic signal in advance of the texture displacement. This possibility would likely depend, however, on some coupling between texture displacement and the time of the following saccade.

The results in Fig. 5D were with texture displacement, and are unlikely to reflect motor programming. Since we cannot train zebrafish to perform prompted saccades (in contrast to experiments with primates), we rely on spontaneously executed saccades, which the fish do at highly variable inter-saccade intervals, ranging from a few seconds up to minutes. It is therefore highly unlikely that the fish attempted to perform anticipatory saccades to texture displacements, when embedded in agarose. The fish could however perform following saccades in response to texture displacements. For data shown in Figure 8B we combined experiments with real saccades and texture displacements within the same animal. For those experiments, fish were able to move their eyes freely, even during texture displacement. We re-inspected that data and found that fish did not perform following saccades directly after or directly before a texture displacement. Thus, there is no saccade-related motor signal available in the texture displacement condition, that could explain pre-saccadic suppression, rendering the proposed possibility highly improbable.

- This lack of understanding for the pre-stimulus period also applies to Figure 1, Figure 7, and Figure 8. In turn, Figure 9 is misleading as in this cartoon the pre-stimulus suppression is always drawn as being the same as that of the baseline at time 8 seconds or later.

As clarified above, we do not think that there is a lack of understanding for the pre-stimulus modulations that we observed. Concerning Figure 9, it is a schematic depiction with no x-axis scaling. This schematic depiction is also not misleading because it does show the presence of pre-saccadic suppression, like we experimentally observed. As we showed in the new Fig. S5B, this pre-saccadic suppression is different from baseline sensitivity, suggesting that the schematic depiction in Fig. 9 is indeed likely to be correct: that is, there is baseline sensitivity that gets suppressed shortly before saccades/texture displacements and then eventually recovers. This is what the schematic shows.

Clarity and writing:

- Figure 6: How many cells? What fraction of the population?

The numbers of cells for each figure are listed in the figure legend. For Figure 6 C & D, cell numbers are listed on the y-axis of the raster plot; for E, the legend states “n = 1,022 ROIs for textured background and shuffled data, 3,274 ROIs for uniform background”; and for F, the legend states “N = 494 ROIs from 6 animals for textured background, n = 643 ROIs from 6 animals for uniform background.” The fraction of neurons used in each experiment is also listed in the Methods section under *Data analysis (Calcium imaging).*

- Figure 7: Unclear how many cells are associated with this figure and if the cells under the different conditions are completely different populations.

The legend for Figure 7 describes the cell numbers for C: “n = 956 ROIs for dot, n = 1,827 ROIs for looming disc” and for D: “N = 312 ROIs from 3 animals for moving dot, n = 145 ROIs from 5 animals for looming disc”. The data shown in this figure were collected from different animals for each condition; therefore, we cannot say with certainty if the neuronal populations are overlapping. However, considering that the stimuli were shown in different locations within visual space (looming dot in upper frontal visual field and looming disc in the lower visual field) and that the two stimuli had vastly different visual features, it is unlikely that there were neurons with receptive fields covering both

stimuli. Förster et al., 2020 have also found non-overlapping populations of tectal neurons for looming stimuli and small moving dot stimuli (see Fig. 2 of Förster et al., 2020 above).

- Many paragraphs are verbose and run-on. At times, figures are presented without reference to the results of each panel, and results are in the figure legends.

We have carefully inspected main text and figure legends regarding the criticism of not properly referencing/explaining individual panels in the text and the criticism of placing results description in the figure legend.

References:

Benisty, H., Song, A., Mishne, G., & Charles, A. S. (2022). Review of data processing of functional optical microscopy for neuroscience. *Neurophotonics*, *9*(4), 041402. https://doi.org/10.1117/1.NPH.9.4.041402

Fornetto, C., Euler, T., & Baden, T. (2025). Zebrafish use spectral information to suppress the visual background. *Cell*, *188*(26), 7512-7528.e13. https://doi.org/10.1016/j.cell.2025.10.009

Förster, D., Helmbrecht, T. O., Mearns, D. S., Jordan, L., Mokayes, N., & Baier, H. (2020). Retinotectal circuitry of larval zebrafish is adapted to detection and pursuit of prey. *ELife*, *9*, 1–26. https://doi.org/10.7554/ELIFE.58596

Idrees, S., Baumann, M. P., Franke, F., Münch, T. A., & Hafed, Z. M. (2020). Perceptual saccadic suppression starts in the retina. *Nature Communications*, *11*(1), 1–19. https://doi.org/10.1038/s41467-020-15890-w

Idrees, S., Baumann, M. P., Korympidou, M. M., Schubert, T., Kling, A., Franke, K., Hafed, Z. M., Franke, F., & Münch, T. A. (2022). Suppression without inhibition: how retinal computation contributes to saccadic suppression. *Communications Biology 2022 5:1*, *5*(1), 1–23. https://doi.org/10.1038/s42003-022-03526-2

Rolls, E. T., Tovée, M. J., & Panzeri, S. (1999). The Neurophysiology of Backward Visual Masking: Information Analysis. *Journal of Cognitive Neuroscience*, *11*(3), 300–311. https://doi.org/10.1162/089892999563409

Shen, T., Lur, G., Xu, X., & Yu, Z. (2022). To deconvolve, or not to deconvolve: Inferences of neuronal activities using calcium imaging data. *Journal of Neuroscience Methods*, *366*, 109431. https://doi.org/10.1016/J.JNEUMETH.2021.109431

Sperling, G. (1965). Temporal and Spatial Visual Masking. I. Masking by Impulse Flashes. *JOSA, Vol. 55, Issue 5, Pp. 541-559*, *55*(5), 541–559. https://doi.org/10.1364/JOSA.55.000541

Wang, K., Hinz, J., Zhang, Y., Thiele, T. R., & Arrenberg, A. B. (2020). Parallel Channels for Motion Feature Extraction in the Pretectum and Tectum of Larval Zebrafish. *Cell Reports*, *30*(2), 442-453.e6. https://doi.org/10.1016/j.celrep.2019.12.031
