# Supplementary material for: Saccadic suppression enhances saliency of ecologically relevant stimuli in the zebrafish optic tectum
Source: PLoS Biol. 2026 Nov 4;24(11):e3004093. doi: 10.1371/journal.pbio.3004093 (PMC13766609; doi:10.1371/journal.pbio.3004093)
Supplement: Supplementary file 5 [file pbio.3004093.s014.docx]

**Response to Reviewer**

Reviewer #3:

The authors have not adequately addressed some important elements of the prior review.

Backward suppression:

A concern that has been raised several times is that key data like that in Figure 5D are being presented without a proper pre-stimulus baseline. This has consistently made it difficult to causally interpret the impact of the texture displacement. The authors have tried to argue that the suppressed pre-stimulus (texture) baseline in Figure 1D and 5D is due to a form of backward masking. However, the evidence they provided in their prior rebuttal for backward masking from Idrees et al. 2022 shows an effect on the 10s of milliseconds timescale, at least an order of magnitude faster than what is being seen here. It is therefore imperative that the authors come to better grips with this issue and demonstrate the role of the texture displacement once a proper pre-stimulus (texture) is established. It will be fine to do this for the behavioral data in Figure 1D. Presumably, given the O-bend response latency, they will need to begin with flashes that start at least 2 seconds ahead of the texture displacement.

As recommended by the editors, we have now explicitly addressed this and the other remaining minor point in the Discussion section of our revised manuscript. In what follows, we briefly explain our position.

In a long stimulus protocol with many trials, the baseline is always (except for the first trial) preceded by a flash or texture displacement. In Figure 5D, the plotted baseline contains trials in which the flash occurred  8 to 12.5 s before the texture displacement. These flashes were not suppressed by backward masking. We acknowledge that we did not record additional pre time steps in the interval of 500 ms to 8 s. However, our data clearly shows that pre-saccadic suppression exists at a time scale of 500 ms, and this pre-saccadic suppression is also behaviorally relevant (see Figure 1). Further investigation of the extended duration of backwards masking is beyond the scope of the manuscript, which focusses on saccadic suppression. Our explanation for the longer time scale in fish compared to mammals is slower neuronal processing and saccade rates in zebrafish, which we discuss in the manuscript. A potential caveat could be the long calcium response decay times (we now added further discussion of this in the manuscript). However, since the behavioral results show a similar time scale of backward masking, the relatively long time scale of backward masking is not caused by the neurophysiology method.

We now write in the discussion section: “*It is to be noted, that due to the slow temporal dynamics of calcium imaging, we cannot with absolute certainty distinguish between backwards masking of the flash response due to texture displacement and forward masking of the texture displacement response due to the pre-saccadic probe flash. Under our analysis conditions, both effects would result in a seemingly suppressed response of the pre-saccadic stimulus. However, we know that backward masking is of behavioral relevance, since O-bend probabilities were reduced for pre-saccadic probe flashes, as can be seen in Fig. 1D.”*

We also already discuss the vast difference in time scale between monkey and zebrafish in lines 587 to 603 in the Discussion section. We have now included comparison of the time scale in the same sentence in order to make sure that the readers do not miss it: “*Another striking difference between saccadic suppression in the zebrafish OT and saccadic suppression in primates is a strong secondary suppression in zebrafish, particularly of global flash responses for several seconds past the saccade onset, while saccadic suppression in the macaque only lasts only for a fraction of a second.* “

And:

*“The observed longer-lasting suppression in zebrafish could potentially be related to the much lower saccade frequencies in zebrafish vs. primates, and further studies are needed to investigate the role of suppression duration and causes for it across species.”*

Fine-texture displacement:

Figure 6F appears to show that there is a significant suppression effect due to the eye movement alone ('No Texture') that is accentuated when the visual feedback is coarsely textured ('Coarse') and greatly reduced when the visual feedback is finely textured ('Fine'). Therefore, one would conclude from this panel that during natural behavior, some textures are actually enhancive and not suppressive relative to a no-texture condition?

In all three conditions, real saccades with coarse visual feedback (“visual & motor combined”, coarse), real saccades with fine visual feedback (“visual & motor combined”, fine), and real saccades without visual feedback (“motor only”), we can see a suppressive effect due to the saccade. Since the suppressive effect is weaker for fine texture than without texture, one could technically interpret this as “enhancive”. However, as shown in Figure 8Bii, we know that different cell populations are suppressed in the “visual & motor combined” condition and the “motor only” condition. Therefore, the effects must be interpreted independently. Fine textures cannot, on their own, “enhance” signals that were suppressed in the “motor only” condition, since the effects take hold on separate neuronal populations. Neurons suppressed in the “visual & motor combined” conditions are not found to be suppressed at all in the “motor only” condition. Thus, fine textures still have a weak suppressive effect.
