# Supplementary material for: Saccadic suppression enhances saliency of ecologically relevant stimuli in the zebrafish optic tectum
Source: PLoS Biol. 2026 Nov 4;24(11):e3004093. doi: 10.1371/journal.pbio.3004093 (PMC13766609; doi:10.1371/journal.pbio.3004093)
Supplement: Supplementary file 2 [file pbio.3004093.s010.docx]

**Response to Reviewers**

Reviewer #1: In this work, Soto and colleagues used ex vivo and in vivo analysis of saccadic suppression in zebrafish. They first recorded from ex vivo retina preparation of adult zebrafish using an MEA and found suppression of visual responses lasting up to 500ms after rapid image transitions. Next, using 2p imaging of the tectum, the authors found extremely prolonged suppression lasting several seconds following texture displacement or actual saccades. Interestingly, unlike global image shifts, localized stimuli were associated with only brief suppression - very different outcome that may hint to fundamental differences in processing of self-induced / global changes and external stimuli with prey/predator statistics. Lastly, the group found that suppression was distinct between different groups of tectal neurons.

I find the results interesting and unexpected. However, I am concerned about potential artifacts in data analysis that could have influenced the interpretation of the experiments

Major points:

1.	I do not understand the difference between probes presented to the fix at -500ms and 8s (Figure 3D). Why are the probes presented before the displacement suppressed? I see three options. Either (1) I am not interpreting the data correctly - in which case, please explain -500ms datapoint better. (2) Perhaps the presentation of texture displacements desensitizes tectal responses ? (3) Bleaching of calcium signals? The latter would indicate a failed experiment.

Option (2) is likely what is happening. The stimulus presented 500ms prior to the texture displacement, which itself is 500 ms long, is likely suppressed due to stimulus-stimulus interactions, such as backwards masking. This phenomenon can also be observed in the context of saccadic suppression in humans in (Idrees, et al. 2020). This is addressed in lines 251-253.

2.	How long does the suppression last in the tectum? The authors only probed it up to 4 seconds - but the suppression seems to be identical over the entire period. Can the suppression last minutes and affect subsequent stimuli? In the example shown in Fig. 4B, some luminance changes are presented with a small delay. How does the history of previous flashes affect flash responses?

We have since tested further delays of 8000, 16000, and 32000ms and have found that responses return to baseline by 8000ms. We have included the results of this experiment in Figure S6 and reported the findings in the Results section under the subheading “Texture displacement suppresses visual responses to global dark flash in the optic tectum” (lines 256ff).

Further we have investigated the effect of a previous flash on the flash responses itself. We did this in two ways: first, we checked how two probe flashes of 500 ms each would interact, and second, we also investigated how a short 30ms flash (more resemblant of a short saccadic event) affects the following probe flash. We have found that when the two flashes in either case are presented shortly after one another, responses to the second flash are minimally smaller. Later delays ≥1000ms are unaffected. This is evidence for a small level of flash-flash interactions, which are likely already processed in the retina. However, we emphasize that these observations cannot fully explain the suppressive effects seen in the tectum, since they are only very weak and have a very short suppressive time window. Two consecutive flash stimuli interact differently than a flash and a texture displacement, as can be seen by smaller suppressive effect of two consecutive flashes. We have included data of the performed flash-flash experiments in Figure S5 and discussed them in terms of visual masking in the discussion section (lines 494ff).

3.	How many animals were used in the in vivo experiments? Were the animals imaged on multiple days or multiple sessions? There are no error bars for the analysis of neuronal populations presented in figure 6. How stable were the responses? Would the same cell remain in the same category between subsequent imaging sessions?

Figure 8A (formerly Fig. 6A) was composed of the data previously shown in Figs. 2E, 5D, 6F, and 7D (formerly Figs. 1E, 3D, 4F, and 5D), in which error bars representing SEM were presented. For Fig. 2E, data came from 10 animals, for Fig. 5D data came from 6 animals, for Fig. 6F data came from 6 animals for textured background experiments and 6 animals for uniform background experiments, for Fig. 7D data came from 3 animals for moving dot experiments and 5 animals for looming disc experiments. In Fig. 8A we chose to omit error bars for easier readability and to avoid convolution of the plot.

For in vivo experiments, each animal was only imaged in one imaging session. In most instances one imaging session consisted only of one stimulus or saccade type experiment, with the exception of data shown in Fig 8B, where within one imaging session, two experiments were conducted. Imaging sessions lasted between 20-90 minutes.

While there can be history-dependent effects and signal bleaching happening, particularly within long imaging sessions, we accounted for those effects by pseudo-randomizing stimulus conditions and correcting signal decay within our analysis by normalizing fluorescence traces with a sliding window median of 120 s (see Methods, Data analysis (Calcium imaging), lines 754-755).

Minor points:

Data presentation:

1.	I had a very hard time understanding the plots shown in Figs. 3B, 4C&D and S2. Was the modulation computed from the peak response? Why do most cells respond to just one delay and were largely unmodulated in others?

Yes, as described in Methods under “Data analysis (Calcium imaging)” (line 770), peak ΔF/F responses were quantified for all trials and modulation was calculated based on peak responses to conditions without texture displacement or saccade. The observation that “most cells respond to just one delay” is in fact an optical illusion due to the data sorting we have done for this type of figure. The modulation in these raster plots is relative to a baseline stimulus condition, shown in the absence of a saccade or texture displacement (which itself is not shown in the raster plot). Cells within lines of the raster plot are first binned by 20^th^, 40^th^, 60^th^, 80^th^ and 100^th^ percentile for percent modulation averaged across all delay times (columns), where cells shown at the top are most suppressed and cells at the bottom of the raster plot are most enhanced, relative to baseline responses. Within each 20^th^ percentile bin, cells are further sorted by the delay where the strongest absolute modulation occurs. The cells are still modulated for other delays, albeit less. This sorting focuses the reader towards seeing trends of peak modulation for different delay times. For example, in Fig 5C and D we can see that there are more cells with strongest modulation for later delays, leading to large vertical bands for 1000, 2000, and 4000ms delays; however, the cells within those rows still have green (suppressed) responses at the other delays. While we agree that this type of plot is complex, we believe it is valuable, because it allows us to visualize modulation strength across cells and delay times while keeping response variability across cells intact.

We now introduce this figure type more clearly in lines (222ff, main text), where we write:

*We sorted cells according to their overall modulation across all flash delays and divided them into 5 bins each carrying 20% of the data. Within each bin, we sorted cells further by their most modulated flash delay in an effort to characterize the relevant delay times for which saccadic suppression or enhancement is present, and to visualize response variation across cells(Figs. 4B & S3).*

We provide further detail on the generation of this plot in the Figure legend of Figure S3:

*1) The overall percent modulation across all delay times is calculated for each cell and cells are sorted in ascending order, 2) all cells are split up in 5 bins (each containing 20% of the cells), and within the bin, neurons are sorted according to the delay time with the strongest modulation (suppression or enhancement), 3) for each peak modulation delay time (within a 20% bin), the corresponding cells are sorted according to their overall suppression or enhancement across all delay times.*

2.	Please change the color of the yellow text / traces on white background in Fig. 4. I couldn't see them well on my screen.

We have updated the font color for better readability. Thank you for pointing that out.

3.	This reviewer would like to see more examples of raw traces and analysis steps. Fig. 2C - it would be great to show zoomed -in traces in addition to the entire experimental time, to better see the kinetics of the probe-displacement interactions.

We include an example of raw calcium data and analysis steps analogous to Fig. 1C in a new Fig. 4 now.

4.	Both course/fine textures are indicated with squares. I propose to change one to another shape.

We have changed all “fine” data point to circles, to easier distinguish them from “coarse” data points.

Technical questions:

1.	Single-unit recordings from a densely grouped RGCs is notoriously difficult. From reading the methods section, it was unclear to me how single units were identified and what were the exclusion criteria (spike shape/firing rate/refractory period etc) to differentiate these from multi units. Perhaps this study did not require single units, but it is important to know how data was selected and curated.

We have now clarified this in Methods under “Data analysis (MEA)” (lines 642-645). Briefly, single units were identified semi-manually by waveform. Waveforms within one unit were first clustered through principal component analysis and clusters were then manually sorted based on waveform variability. Clusters with unreasonable inter-spike times or large waveform variability were excluded.

2.	What was the refresh rate of the projector?

For electrophysiology recordings, visual stimuli were presented on a DLP projector with a refresh rate of 60 Hz. We have now included this information in the Methods section under “Visual stimulation (MEA)” (line 613).

Reviewer #2: In this study, Soto et al. used multi-electrode array (MEA) recordings of adult zebrafish retinal explants to show texture displacement could suppress visual responses of a second visual stimulus within 300 ms. They then switched to larval zebrafish calcium imaging and observed a surprisingly long-lasting saccade-mediated suppression in Optic Tectum neurons. Interestingly, the duration and amplitude of suppression is much higher for global vs. local stimuli, and different populations of tectal neurons are suppressed by the sensory vs motor saccade signal.

This study is well-designed and executed, and achieves the following goals 1. Demonstration that saccadic suppression occurs in larval zebrafish. 2. Identification and characterization of the subcortical neural population subject to saccadic suppression and visual masking, 3. Delineation of the visual feedback and motor contributions. That said, there are still conceptual gaps which require further experimental investigations. I would recommend the paper to be accepted after the key issues are addressed.

Major points:

1.	Currently, there is no behavioral data to indicate whether larvae responded to the dark dot and flash stimuli. Do these stimuli trigger startle or hunting in the authors' setup, and what percentage of the time? 20° diameter dark dots are not the optimal stimuli for hunting, so unless they are actually triggering hunting they should be called "local" instead of "prey-like" stimuli in the title and throughout the text. If the stimuli do induce hunting or startle behavior, then the response should be analysed, as it may affect the results.

We are aware that 20° dots, especially without a UV component, are not ideal for evoking hunting behavior. Prey in the periphery is perceived by the fish as a much smaller object, usually modeled as a 5° in the literature (Bianco et al., 2011; Khan et al., 2023; Semmelhack et al., 2014). On the other hand, prey in the capture strike zone is perceived as a much larger object, usually modeled as a 30° dot (Förster et al., 2020). We wanted to strike a balance between a large enough object that would activate multiple neurons, while also ensuring a small enough object so that local processing structures mattered. Therefore, we chose an intermediately sized object of 20°, which moved back and forth in the lateral visual field between 0° and 60° azimuth. This represents a “prey-like” stimulus as the fish is orienting and swimming towards the prey.

Concerning behavioral data, this is indeed an interesting idea. However, we feel that it is beyond the scope of the current paper. In follow up work, we will certainly make use of our findings to investigate the behavioral consequences of the selective nature of saccadic suppression that we have uncovered here. Please also see the response to the next comment about looming.

2.	If saliency filtering is the underlying function for cue specific OT suppression duration, it would be informative to perform other ethological relevant cues such as looming, to see if suppression to looming stimulus is also brief.

We have tested a looming disc stimulus in tectal recordings and have found that suppression for this stimulus also only lasts very briefly. This further supports our interpretation that late saccadic suppression is used as a saliency filter. Ecologically more relevant stimuli thus as moving dots (hunting) and looming discs (escape response) are suppressed less than an ecologically less relevant stimulus (global flash, which should occur frequently in natural habitats due to water surface waves). We have included the looming disc data in Figure 5 and changed the manuscript title slightly.

3.	Motor saccade suppression level and duration for prey stimulus was not performed; it would be important to know how persistently and strongly a more naturalistic saccade (motor + sensory feedback) would suppress the prey response in the OT.

While this would be an excellent experiment to see if ecologically relevant stimuli are also less suppressed with a naturalistic saccade, we are unfortunately technically limited with our current closed-loop setup. In order to measure consistent neural responses, regardless of the eye position of the fish, stimulus presentation needs to take eye position into account. With our current setup, we are limited to horizontal eye position and cannot consider torsional or vertical changes in eye position. Furthermore, in larval zebrafish, eye position does not remain constant after saccadic eye movements. Instead, the eyes quickly drift back to neutral due to a leaky position command in the hindbrain (Brysch et al., 2019). This makes accurate stimulus presentation across trials for local stimuli unfortunately impossible.

4.	There is so far no neural mechanism proposed to explain 1. How ethological relevant cues are differentially suppressed and 2. How saccadic suppression is prolonged in the OT. 3. What is the adaptive value of differential sensory and motor suppression onto distinct OT neurons. I would appreciate an exploration of these questions in the discussion, without more experiments.

We have now added a discussion regarding the possible mechanism in lines 610-618. To keep our manuscript concise, we did not speculate regarding the prolongation mechanism, and regarding the differential sensory vs. motor suppression.

5.	In the introduction, the authors propose zebrafish as a model system to study saccade suppression, so it would be helpful to explain the obstacles to progress in understanding this question in primates, and how using zebrafish can solve these issues.

We believe that accessing zebrafish as a model organism for saccadic suppression has many benefits. For one, testing principles in small brains allows us to constrain computational model complexities that account for saccadic suppression. Secondly, we are quite interested in comparative biology between different species (see our recent preprint (Soto et al., 2025)). And lastly, we believe species crosstalk can lead to interesting predictions in primates. For instance, the selective nature of suppression found in zebrafish, which we discovered in this work, has prompted us to investigate selective saccadic suppression in primates, where we are currently preparing a manuscript.

Minor Points：

1.	In general, the figure labels are too small to read on A4 pages.

Labels have been enlarged, where possible.

2.	Fig.S1 (A) RF size and Response latency error bars are not clearly visible

The color of the data points has been changed to grey. This way, the mean values and error bars in black are more visible.

3.	Line 115-118 should be Fig. S1 instead Fig. S2

Corrected.

4.	Fig 2: Would texture displacement induce saccade motion in larval zebrafish? If so, how does it influence the activity readout of texture displacement related activity and post-displacement suppression?

When inspecting video recordings of embedded fish, it can be seen that while the fish can make minimal eye movements (<5°) even when embedded, the small eye movements are independent of texture displacement presentation and should therefore not cause a systematic effect on post-displacement suppression.

Reviewer #3: In this work, Soto and colleagues demonstrate that a) cells in the zebrafish show a suppression of flash responses at short-time scales following saccade-like displacements of the visual scene, b) that cells in the downstream tectum show a much longer duration suppression of flash responses that is related to eye movement, and that c) this long duration suppression in the tectum is absent during responses to moving prey. The latter two findings, if fully characterized, would be highly novel and suggest a mechanism for enhancement of visual saliency. However, there are major revisions needed to elucidate the relationship between retinal and tectal activity and fully characterize the tectal data.

Comparing RGC and tectal activity:

The authors first assess saccadic suppression in the retina to serve as a starting point for understanding suppression in the tectum. However, while there are strong texture displacement responses in the retina on the timescale of the suppression seen in the retina, there appears to be no or little texture displacement responses in the tectum (Figure 1C). The authors should quantify the presence or lack of texture displacement responses in the tectum. If indeed there is no comparable response, this immediately suggests that any suppressive mechanisms in the tectum will be independent from those in the retina.

We have found small neural responses to both texture displacement and naturalistic saccades within the tectum. However, we still believe, as suggested by Reviewer #3, that suppressive mechanisms within the tectum are independent of those observed in the retina. At the very least, there are likely additional mechanisms at play in the tectum that are not present in retina. Data for texture displacement and real saccade responses can now be found in Figure 3D.

Please also note a lack of tectal response to saccade displacements is itself an interesting phenomenon. In parallel work, we are comparing the texture displacement response in primate V1 and superior colliculus (SC), and we also see a lack of saccade-induced response in the SC (with real saccades). This is suggesting that the SC (and OT in fish) actively cancels saccade-induced image shifts, which may be related to the phenomenon of saccadic omission. Then, if there is a flash, like in our paradigms, the flash response is itself suppressed. We have discussed these two related phenomena (saccadic omission and saccadic suppression of flash responses) in a recent theoretical investigation related to our work (Hafed et al., 2025).

While the raw data supporting the suppressive effect the tectum is nicely presented in Figure 1D, there is no comparable figure for the tectal responses. Raw data from the tectum should be presented that support the finding of saccadic suppression (for both texture displacement and real saccades).

A comparable figure for Ca-data can now be found in a new figure, Fig. 4.

In addition, while suppression in the retina is studied with ~20 ms luminance changes, that in the tectum in the tectum is studied primarily with a slowly varying luminance change over 500 ms. This of course raises concern that we are looking at different physiological responses, which is however mostly addressed by the smaller imaging subset in supplemental where 20 ms luminance changes are used. It is recommended that the 20 ms data be given more prominence early on.

We now also mention the 20 ms data in the Results section under the subheading “Texture displacement suppresses visual responses to global dark flash in the optic tectum” (lines 263ff).

Additionally, all of the tectal analysis is done using calcium signals, which makes it difficult to compare with the retinal data acquired using ephys. The signals should be compared in the same domain.

While electrophysiological data allows for much greater temporal resolution, it is difficult to perform electrophysiological recordings in the tectum of behaving larval zebrafish. Calcium imaging on the other hand allows us to record thousands of neurons non-invasively, allowing the fish to perform natural behaviors unbothered by recording probes. We are aware of the comparative limitations between zebrafish retina and tectum data. However, we believe to have struck a good balance, by opting for temporal resolution and methodical comparativeness between retinal recordings of zebrafish explants in this study and mammalian explants in previous studies. We also believe that it was critical for us to perform the retina experiments, especially given the amazing correspondence with earlier retinal electrophysiology results with mice, pigs, and primates (Idrees et al., 2020, 2022). Whereas in tectal recordings we opted for large scale Ca-imaging at the cost of temporal resolution, to allow us to non-invasively find trends in a big data set. Future studies could examine RGC responses in the neuropil of the tectum with calcium imaging techniques for better comparativeness. We have now added a sentence early in the results section to mention this issues and explain why we performed the experiments in this way (l.173ff): *In theory, it would be ideal to compare retina and tectum at the identical developmental stages using the same activity recording technique, but due to technical limitations our comparison is limited to adult retina electrophysiology and larval tectum calcium imaging.*

Comparison to shuffled data:

The analysis involving shuffling is not clearly explained. For example, for Figure 3B, what are the time bins used for the shuffling? Is shuffling occurring over just the ~5 seconds shown or a longer time series? Is cell identity maintained as we compare the shuffled data at right to the original data at left?

For all raster plots (Figs 5B, 6C, 6D, and S2) and cumulative distribution plots (Figs 5A, 6E, and 7C), data was shuffled by randomly assigning delay identities to each trial. Cell identities were maintained during shuffling. This is now also clarified in the respective figure legends, and briefly in the main text. After shuffling, the raster plots were sorted as described in supplementary figure legend S3 separately for real data and shuffled data. This means that the first line of the raster plot on the left does not necessarily correspond to the same cell in line 1 of the right raster plot.

Additionally, comparing to shuffled calcium data very likely masks the significance of the difference in underlying firing rates. Shuffling of sparse but heavily filtered activity will lead to permuted data that is much closer to the original that shuffling of unfiltered data. Comparison to shuffled data would be much more informative in the deconvolved, firing rate domain.

As mentioned above, delay identities to the extracted responses were shuffled, not the signal trace itself, rendering this suggestion unnecessary.

Characterization of the suppressive effect in the tectum:

The authors have not fully characterized the suppressive signal in the tectum.

In Figure 3D, it is noted that there is a suppression even before the displacement, motivating investigation of motor effects that could contribute. However, when presenting the suppressive signal associated with real saccades, the pre-saccadic suppression is not measured or commented upon (4F, 5D). The authors should present any pre-saccadic effects and do so in a manner that takes into account the time from the previous saccade.

Since the fish perform spontaneous saccades with very long and highly variable inter-saccade intervals, it is impossible for us to predict an upcoming saccade. Therefore, we cannot effectively present a stimulus in the relevant perisaccadic window prior to a spontaneous saccade. The spontaneous saccade rate is so low that it’s very difficult to get enough data.

Related, the authors also do not characterize how long the post-saccadic suppressive signal lasts. One can infer that it lasts somewhere between 4 and 8 seconds, but this should be made clear.

As mentioned above in response to Reviewer #1, we have since tested further delays of 8000, 16000, and 32000ms and have found that responses return to baseline by 8000ms. We have included the results of this experiment in Figure S5.

In addition, given the duration of the tectal suppression, the authors should consider that this effect is more related to eye position and not the saccadic event itself. The authors have data to disambiguate.

We checked whether post-saccadic eye eccentricity had an effect on flash response modulation. A Spearman rank correlation across >111,000 saccades revealed a negligible effect size of rho < -0.01. We conclude that eye position has no effect on saccadic suppression. This conclusion is further supported by the fact that we can see saccadic suppression in simulated saccades, where there is no change in eye position.

Minor:

The numbering/referencing of the supplemental figures from the main text is often misdirected.

We have fixed those errors. Thank you for alerting us.

**References:**

Bianco, I. H., Kampff, A. R., & Engert, F. (2011). Prey capture behavior evoked by simple visual stimuli in larval zebrafish. *Frontiers in Systems Neuroscience*, *5*(DECEMBER 2011), 18496. https://doi.org/10.3389/FNSYS.2011.00101/BIBTEX

Brysch, C., Leyden, C., & Arrenberg, A. B. (2019). Functional architecture underlying binocular coordination of eye position and velocity in the larval zebrafish hindbrain. *BMC Biology*, *17*(1), 1–22. https://doi.org/10.1186/S12915-019-0720-Y/TABLES/1

Förster, D., Helmbrecht, T. O., Mearns, D. S., Jordan, L., Mokayes, N., & Baier, H. (2020). Retinotectal circuitry of larval zebrafish is adapted to detection and pursuit of prey. *ELife*, *9*, 1–26. https://doi.org/10.7554/ELIFE.58596

Hafed, Z. M., Arrenberg, A., Schwarz, C., Benda, J., & Grewe, J. (2025). Statistical regularities and the sensory consequences of self-action: A multi-species, multi-modal perspective. *Current Opinion in Neurobiology*, *94*, 103087. https://doi.org/10.1016/J.CONB.2025.103087

Idrees, S., Baumann, M. P., Franke, F., Münch, T. A., & Hafed, Z. M. (2020). Perceptual saccadic suppression starts in the retina. *Nature Communications*, *11*(1), 1–19. https://doi.org/10.1038/s41467-020-15890-w

Idrees, S., Baumann, M. P., Korympidou, M. M., Schubert, T., Kling, A., Franke, K., Hafed, Z. M., Franke, F., & Münch, T. A. (2022). Suppression without inhibition: how retinal computation contributes to saccadic suppression. *Communications Biology 2022 5:1*, *5*(1), 1–23. https://doi.org/10.1038/s42003-022-03526-2

Khan, B., Jaesiri, O. mongkol, Lazarte, I. P., Li, Y., Tian, G., Zhao, P., Zhao, Y., Ho, V. D., & Semmelhack, J. L. (2023). Zebrafish larvae use stimulus intensity and contrast to estimate distance to prey. *Current Biology*, *33*(15), 3179-3191.e4. https://doi.org/10.1016/j.cub.2023.06.046

Semmelhack, J. L., Donovan, J. C., Thiele, T. R., Kuehn, E., Laurell, E., & Baier, H. (2014). A dedicated visual pathway for prey detection in larval zebrafish. *ELife*, *3*. https://doi.org/10.7554/ELIFE.04878

Soto, G., Dehmelt, F. A., Baumann, M. P., Tunç, I., Yu, Y., Malevich, T., Hafed, Z. M., & Arrenberg, A. B. (2025). Species-specific oculomotor control tolerances predict saccadic suppression strength in macaques and zebrafish. *BioRxiv*, 2025.06.09.658731. https://doi.org/10.1101/2025.06.09.658731
