# Supplementary material for: Saccadic suppression enhances saliency of ecologically relevant stimuli in the zebrafish optic tectum
Source: PLoS Biol. 2026 Nov 4;24(11):e3004093. doi: 10.1371/journal.pbio.3004093 (PMC13766609; doi:10.1371/journal.pbio.3004093)
Supplement: Supplementary file 3 [file pbio.3004093.s012.docx]

**Responses to Reviewer 3**


Reviewer #3: The authors have done a fairly thorough job responding to prior comments. A few remaining points:

Backward suppression

If as described, this is indeed quite interesting. To get a sense of how long a time-window this phenomenon covers in the zebrafish, the authors should perform behavioral experiments as done in Figure 1 and carefully determine at what delay between flash and texture displacement the effect goes away. Neural activity recordings would of course be helpful but do not appear to be necessary.

Backward masking is an interesting and well-described phenomenon of visual stimulus-stimulus interactions. However, it is not the primary focus of this study, so the requested data would be beyond the scope of our current study. Future work is needed to characterize the time course of the effect in zebrafish in more detail and to fully understand the underlying mechanisms. Having said that, to better place the results of our current study within the context of the current literature on neural and behavioral backward masking, we have now included a dedicated paragraph within the Discussion section regarding backward masking. We now write:

*“Interestingly, saccadic suppression was not restricted to probe stimuli following texture displacement, but also occurred when probe stimuli were shown prior to the texture displacement. This phenomenon, called backward masking, was only found in the OT and not in retinal recordings. However, it is of behavioral relevance, since O-bend probabilities were reduced for pre-saccadic probe flashes, as can be seen in Fig. 1D. While backwards masking has been described extensively in mammalian vision research, including some of our own work [6,38–40], the exact underlying mechanisms have not been uncovered. Possibilities include a) slower arrival of flash responses than texture displacement responses within the OT, b) the role of the cessation of the flash, rather than its onset, and c) slow feedback circuits from other brain areas to the OT. Future studies will have to disentangle these possibilities.“*

Regarding a lack of coupling between texture displacement and saccades in Figure 8B: the authors should show this data somewhere. They should also take care to avoid language that implies just because the eyes were embedded there was no motor signal.

We have now measured the mean of the absolute eye position change (Δ|θ|) from 1 sec prior to texture displacement onset to 1 sec after texture displacement and then compared Δ|θ| for CW and CCW texture displacements to a null distribution generated by circularly permuting eye traces 5000 times (permutation = random shift in time of the whole eye trace relative to the stimulus time trace). There was no significant change in eye position during texture displacement (see figures below, p = 0.16 for CW texture displacements, p = 0.73 for CCW texture displacements). We have included this information in the Methods section (see lines 777 to 787) of the revised manuscript and briefly report these results early on in the Results section: “*Separate experiments (see Methods) showed that animals do not perform saccades in response to texture displacement. Thus (in most of our trials) our texture displacement was not associated with a saccadic motor signal (p = 0.73 for a permutation test on saccade probability around the time point of counter-clockwise texture displacement and p = 0.16 for clockwise texture displacements).*”

We also checked the entire manuscript again and removed instances in which we claimed that motor signals were not present at all. We adjusted the wording to say that “for most trials” there should not have been any saccade (given the results of our statistical test above) and kept the statement that the texture displacement corresponds to a stimulus, which in most trials is not associated with a saccadic motor command.

Figure 1. Null-distribution of mean absolute change in eye position (|Δθ|) during CW and CCW texture displacement, obtained by circularly permuting eye traces 5000 times. Red lines indicate two-tailed significance cut-offs, where p < 0.025. Green lines indicate observed |Δθ|.

Text in Discussion is warranted on backward suppression. In doing so, the authors should also try to explain why no backward suppression is seen in Figure S5B.

We have now included backward masking in our discussion as described above.

For Fig. S5B, we can see that there is significant backward suppression, albeit at a smaller level than what we saw in our other data set presented in Fig. 5D, which was done with the same experimental paradigm. The apparent difference in effect size could partially be attributed to the fact that only 3 animals were recorded for the data shown in Fig. S5B (compared to the 6 animals in Fig. 5D); thus, the effect could be less obvious. However, since the mechanisms of pre-saccadic suppression/backward masking are not well understood, it is difficult to speculate on exact reasons. A more comprehensive understanding of the mechanisms of backward masking should be the goal of future studies.

Fine-texture displacement

The effect of fine texture appears to be quite variable: it looks to be suppressive in Figure 5D, but not so in Figure S5C, and could be interpreted as enhancing in Figure 6F. This variability should be noted and discussed.

We are not sure that we understand this comment. Our data in Fig. 5D and 6F show that both the fine texture displacement and saccades across a finely textured background led to significant suppression, and for up to 4 sec. In both cases, suppression was weaker for fine textures compared to the coarse patterns. However, there was no enhancing effect of the fine textures, since suppression was still significant. As for Fig. S5C, here we used a rather small dataset, consisting of only 30 ROIs, making the small effect of fine textures hard to interpret amongst noise.

Cell numbers

The point about 2% responders and the possibility of other circuits contributing to the behavior suppression should be in the Discussion.

To inform readers not familiar with the zebrafish vision literature, we now mention in the results section that the percentage of 2 % is consistent with the literature. We write:

*“While the proportion of flash responsive ROIs, relative to all segmented ROIs seems rather small, it is consistent with previous findings [34]. The optic tectum has very diverse visual receptive fields. Global probe flashes likely mostly activated large receptive fields [35] and we only presented dark flashes, further reducing the fraction of responsive ROIs.”*

Legends

The legends still contain many results that should be part of the main text.

The results of every figure panel have been mentioned at least once in the text. In some cases, we have consciously refrained from putting statistical test results within the main text and only include them within the legend for better readability.
