# Supplementary material for: Saccadic suppression enhances saliency of ecologically relevant stimuli in the zebrafish optic tectum
Source: PLoS Biol. 2026 Nov 4;24(11):e3004093. doi: 10.1371/journal.pbio.3004093 (PMC13766609; doi:10.1371/journal.pbio.3004093)
Supplement: Peer review history

# Editor report: Decision Letter 0

**DOI:** 10.1371/journal.pbio.3004093.r001

**Authors:** Taylor Hart, PhD

9 Sep 2025

Dear Dr Arrenberg,

Thank you for submitting your manuscript entitled "Saccadic suppression enhances saliency of prey-like stimuli in the optic tectum" for consideration as a Research Article by PLOS Biology.

Your manuscript has now been evaluated by the PLOS Biology editorial staff, as well as by an academic editor with relevant expertise, and I am writing to let you know that we would like to send your submission out for external peer review.

However, before we can send your manuscript to reviewers, we need you to complete your submission by providing the metadata that is required for full assessment. To this end, please login to Editorial Manager where you will find the paper in the 'Submissions Needing Revisions' folder on your homepage. Please click 'Revise Submission' from the Action Links and complete all additional questions in the submission questionnaire.

Once your full submission is complete, your paper will undergo a series of checks in preparation for peer review. After your manuscript has passed the checks it will be sent out for review. To provide the metadata for your submission, please Login to Editorial Manager (https://www.editorialmanager.com/pbiology) within two working days, i.e. by Sep 11 2025 11:59PM.

If your manuscript has been previously peer-reviewed at another journal, PLOS Biology is willing to work with those reviews in order to avoid re-starting the process. Submission of the previous reviews is entirely optional and our ability to use them effectively will depend on the willingness of the previous journal to confirm the content of the reports and share the reviewer identities. Please note that we reserve the right to invite additional reviewers if we consider that additional/independent reviewers are needed, although we aim to avoid this as far as possible. In our experience, working with previous reviews does save time.

If you would like us to consider previous reviewer reports, please edit your cover letter to let us know and include the name of the journal where the work was previously considered and the manuscript ID it was given. In addition, please upload a response to the reviews as a 'Prior Peer Review' file type, which should include the reports in full and a point-by-point reply detailing how you have or plan to address the reviewers' concerns.

During the process of completing your manuscript submission, you will be invited to opt-in to posting your pre-review manuscript as a bioRxiv preprint. Visit http://journals.plos.org/plosbiology/s/preprints for full details. If you consent to posting your current manuscript as a preprint, please upload a single Preprint PDF.

Feel free to email us at plosbiology@plos.org if you have any queries relating to your submission.

Kind regards,

Taylor

Taylor Hart, PhD,

Associate Editor

PLOS Biology

thart@plos.org

# Peer review: Decision Letter 1

**DOI:** 10.1371/journal.pbio.3004093.r002

**Authors:** Taylor Hart, PhD

31 Oct 2025

Dear Dr Arrenberg,

Thank you for your patience while your manuscript "Saccadic suppression enhances saliency of prey-like stimuli in the optic tectum" was peer-reviewed at PLOS Biology. It has now been evaluated by the PLOS Biology editors, an Academic Editor with relevant expertise, and by several independent reviewers.

In light of the reviews, which you will find at the end of this email, we would like to invite you to revise the work to thoroughly address the reviewers' reports.

As you will see, the reviewers wrote that the results are quite interesting and potentially novel. However, each of them pointed out gaps in the experiments and analyses that raise doubts about some of the interpretation of the data. In your revision, you should carefully consider the reviewers' points and address them thoroughly.

Given the extent of revision needed, we cannot make a decision about publication until we have seen the revised manuscript and your response to the reviewers' comments. Your revised manuscript is likely to be sent for further evaluation by all or a subset of the reviewers.

In addition to these revisions, you will need to complete some formatting changes, which you will receive in a follow up email. A member of our team will be in touch with a set of requests shortly.

We expect to receive your revised manuscript within 3 months. Please email us (plosbiology@plos.org) if you have any questions or concerns, or would like to request an extension.

At this stage, your manuscript remains formally under active consideration at our journal; please notify us by email if you do not intend to submit a revision so that we may withdraw it.

**IMPORTANT - SUBMITTING YOUR REVISION**

Your revisions should address the specific points made by each reviewer. Please submit the following files along with your revised manuscript:

1. A 'Response to Reviewers' file - this should detail your responses to the editorial requests, present a point-by-point response to all of the reviewers' comments, and indicate the changes made to the manuscript.

*NOTE: In your point-by-point response to the reviewers, please provide the full context of each review. Do not selectively quote paragraphs or sentences to reply to. The entire set of reviewer comments should be present in full and each specific point should be responded to individually, point by point.

You should also cite any additional relevant literature that has been published since the original submission and mention any additional citations in your response.

2. In addition to a clean copy of the manuscript, please also upload a 'track-changes' version of your manuscript that specifies the edits made. This should be uploaded as a "Revised Article with Changes Highlighted" file type.

*Re-submission Checklist*

When you are ready to resubmit your revised manuscript, please refer to this re-submission checklist: https://plos.io/Biology_Checklist

To submit a revised version of your manuscript, please go to https://www.editorialmanager.com/pbiology/ and log in as an Author. Click the link labelled 'Submissions Needing Revision' where you will find your submission record.

Please make sure to read the following important policies and guidelines while preparing your revision:

*Published Peer Review*

Please note while forming your response, if your article is accepted, you may have the opportunity to make the peer review history publicly available. The record will include editor decision letters (with reviews) and your responses to reviewer comments. If eligible, we will contact you to opt in or out. Please see here for more details:

https://blogs.plos.org/plos/2019/05/plos-journals-now-open-for-published-peer-review/

*PLOS Data Policy*

Please note that as a condition of publication PLOS' data policy (http://journals.plos.org/plosbiology/s/data-availability) requires that you make available all data used to draw the conclusions arrived at in your manuscript. If you have not already done so, you must include any data used in your manuscript either in appropriate repositories, within the body of the manuscript, or as supporting information (N.B. this includes any numerical values that were used to generate graphs, histograms etc.). For an example see here: http://www.plosbiology.org/article/info%3Adoi%2F10.1371%2Fjournal.pbio.1001908#s5

*Blot and Gel Data Policy*

We require the original, uncropped and minimally adjusted images supporting all blot and gel results reported in an article's figures or Supporting Information files. We will require these files before a manuscript can be accepted so please prepare them now, if you have not already uploaded them. Please carefully read our guidelines for how to prepare and upload this data: https://journals.plos.org/plosbiology/s/figures#loc-blot-and-gel-reporting-requirements

*Protocols deposition*

To enhance the reproducibility of your results, we recommend that if applicable you deposit your laboratory protocols in protocols.io, where a protocol can be assigned its own identifier (DOI) such that it can be cited independently in the future. Additionally, PLOS ONE offers an option for publishing peer-reviewed Lab Protocol articles, which describe protocols hosted on protocols.io. Read more information on sharing protocols at https://plos.org/protocols?utm_medium=editorial-email&utm_source=authorletters&utm_campaign=protocols

Thank you again for your submission to our journal. We hope that our editorial process has been constructive thus far, and we welcome your feedback at any time. Please don't hesitate to contact us if you have any questions or comments.

Sincerely,

Taylor

Taylor Hart, PhD,

Associate Editor

PLOS Biology

thart@plos.org

------------------------------------

REVIEWS:

Reviewer #1: In this work, Soto and colleagues used ex vivo and in vivo analysis of saccadic suppression in zebrafish. They first recorded from ex vivo retina preparation of adult zebrafish using an MEA and found suppression of visual responses lasting up to 500ms after rapid image transitions. Next, using 2p imaging of the tectum, the authors found extremely prolonged suppression lasting several seconds following texture displacement or actual saccades. Interestingly, unlike global image shifts, localized stimuli were associated with only brief suppression - very different outcome that may hint to fundamental differences in processing of self-induced / global changes and external stimuli with prey/predator statistics. Lastly, the group found that suppression was distinct between different groups of tectal neurons.

I find the results interesting and unexpected. However, I am concerned about potential artifacts in data analysis that could have influenced the interpretation of the experiments

Major points:

1.I do not understand the difference between probes presented to the fix at -500ms and 8s (Figure 3D). Why are the probes presented before the displacement suppressed? I see three options. Either (1) I am not interpreting the data correctly - in which case, please explain -500ms datapoint better. (2) Perhaps the presentation of texture displacements desensitizes tectal responses ? (3) Bleaching of calcium signals? The latter would indicate a failed experiment.

2.How long does the suppression last in the tectum? The authors only probed it up to 4 seconds - but the suppression seems to be identical over the entire period. Can the suppression last minutes and affect subsequent stimuli? In the example shown in Fig. 4B, some luminance changes are presented with a small delay. How does the history of previous flashes affect flash responses?

3.How many animals were used in the in vivo experiments? Were the animals imaged on multiple days or multiple sessions? There are no error bars for the analysis of neuronal populations presented in figure 6. How stable were the responses? Would the same cell remain in the same category between subsequent imaging sessions?

Minor points:

Data presentation:

1.I had a very hard time understanding the plots shown in Figs. 3B, 4C&D and S2. Was the modulation computed from the peak response? Why do most cells respond to just one delay and were largely unmodulated in others?

2.Please change the color of the yellow text / traces on white background in Fig. 4. I couldn't see them well on my screen.

3.This reviewer would like to see more examples of raw traces and analysis steps. Fig. 2C - it would be great to show zoomed -in traces in addition to the entire experimental time, to better see the kinetics of the probe-displacement interactions.

4.Both course/fine textures are indicated with squares. I propose to change one to another shape.

Technical questions:

1.Single-unit recordings from a densely grouped RGCs is notoriously difficult. From reading the methods section, it was unclear to me how single units were identified and what were the exclusion criteria (spike shape/firing rate/refractory period etc) to differentiate these from multi units. Perhaps this study did not require single units, but it is important to know how data was selected and curated.

2.What was the refresh rate of the projector?

Reviewer #2: In this study, Soto et al. used multi-electrode array (MEA) recordings of adult zebrafish retinal explants to show texture displacement could suppress visual responses of a second visual stimulus within 300 ms. They then switched to larval zebrafish calcium imaging and observed a surprisingly long-lasting saccade-mediated suppression in Optic Tectum neurons. Interestingly, the duration and amplitude of suppression is much higher for global vs. local stimuli, and different populations of tectal neurons are suppressed by the sensory vs motor saccade signal.

This study is well-designed and executed, and achieves the following goals 1. Demonstration that saccadic suppression occurs in larval zebrafish. 2. Identification and characterization of the subcortical neural population subject to saccadic suppression and visual masking, 3. Delineation of the visual feedback and motor contributions. That said, there are still conceptual gaps which require further experimental investigations. I would recommend the paper to be accepted after the key issues are addressed.

Major points:

1.Currently, there is no behavioral data to indicate whether larvae responded to the dark dot and flash stimuli. Do these stimuli trigger startle or hunting in the authors' setup, and what percentage of the time? 20° diameter dark dots are not the optimal stimuli for hunting, so unless they are actually triggering hunting they should be called "local" instead of "prey-like" stimuli in the title and throughout the text. If the stimuli do induce hunting or startle behavior, then the response should be analysed, as it may affect the results.

2.If saliency filtering is the underlying function for cue specific OT suppression duration, it would be informative to perform other ethological relevant cues such as looming, to see if suppression to looming stimulus is also brief.

3.Motor saccade suppression level and duration for prey stimulus was not performed; it would be important to know how persistently and strongly a more naturalistic saccade (motor + sensory feedback) would suppress the prey response in the OT.

4.There is so far no neural mechanism proposed to explain 1. How ethological relevant cues are differentially suppressed and 2. How saccadic suppression is prolonged in the OT. 3. What is the adaptive value of differential sensory and motor suppression onto distinct OT neurons. I would appreciate an exploration of these questions in the discussion, without more experiments.

5.In the introduction, the authors propose zebrafish as a model system to study saccade suppression, so it would be helpful to explain the obstacles to progress in understanding this question in primates, and how using zebrafish can solve these issues.

Minor Points：

1.In general, the figure labels are too small to read on A4 pages.

2.Fig.S1 (A) RF size and Response latency error bars are not clearly visible

3.Line 115-118 should be Fig. S1 instead Fig. S2

4.Fig 2: Would texture displacement induce saccade motion in larval zebrafish? If so, how does it influence the activity readout of texture displacement related activity and post-displacement suppression?

Reviewer #3: In this work, Soto and colleagues demonstrate that a) cells in the zebrafish show a suppression of flash responses at short-time scales following saccade-like displacements of the visual scene, b) that cells in the downstream tectum show a much longer duration suppression of flash responses that is related to eye movement, and that c) this long duration suppression in the tectum is absent during responses to moving prey. The latter two findings, if fully characterized, would be highly novel and suggest a mechanism for enhancement of visual saliency. However, there are major revisions needed to elucidate the relationship between retinal and tectal activity and fully characterize the tectal data.

Comparing RGC and tectal activity:

The authors first assess saccadic suppression in the retina to serve as a starting point for understanding suppression in the tectum. However, while there are strong texture displacement responses in the retina on the timescale of the suppression seen in the retina, there appears to be no or little texture displacement responses in the tectum (Figure 1C). The authors should quantify the presence or lack of texture displacement responses in the tectum. If indeed there is no comparable response, this immediately suggests that any suppressive mechanisms in the tectum will be independent from those in the retina.

While the raw data supporting the suppressive effect the tectum is nicely presented in Figure 1D, there is no comparable figure for the tectal responses. Raw data from the tectum should be presented that support the finding of saccadic suppression (for both texture displacement and real saccades).

In addition, while suppression in the retina is studied with ~20 ms luminance changes, that in the tectum in the tectum is studied primarily with a slowly varying luminance change over 500 ms. This of course raises concern that we are looking at different physiological responses, which is however mostly addressed by the smaller imaging subset in supplemental where 20 ms luminance changes are used. It is recommended that the 20 ms data be given more prominence early on.

Additionally, all of the tectal analysis is done using calcium signals, which makes it difficult to compare with the retinal data acquired using ephys. The signals should be compared in the same domain.

Comparison to shuffled data:

The analysis involving shuffling is not clearly explained. For example, for Figure 3B, what are the time bins used for the shuffling? Is shuffling occurring over just the ~5 seconds shown or a longer time series? Is cell identity maintained as we compare the shuffled data at right to the original data at left?

Additionally, comparing to shuffled calcium data very likely masks the significance of the difference in underlying firing rates. Shuffling of sparse but heavily filtered activity will lead to permuted data that is much closer to the original that shuffling of unfiltered data. Comparison to shuffled data would be much more informative in the deconvolved, firing rate domain.

Characterization of the suppressive effect in the tectum:

The authors have not fully characterized the suppressive signal in the tectum.

In Figure 3D, it is noted that there is a suppression even before the displacement, motivating investigation of motor effects that could contribute. However, when presenting the suppressive signal associated with real saccades, the pre-saccadic suppression is not measured or commented upon (4F, 5D). The authors should present any pre-saccadic effects and do so in a manner that takes into account the time from the previous saccade.

Related, the authors also do not characterize how long the post-saccadic suppressive signal lasts. One can infer that it lasts somewhere between 4 and 8 seconds, but this should be made clear.

In addition, given the duration of the tectal suppression, the authors should consider that this effect is more related to eye position and not the saccadic event itself. The authors have data to disambiguate.

Minor:

The numbering/referencing of the supplemental figures from the main text is often misdirected.

# Author comment: Author response to Decision Letter 2

**DOI:** 10.1371/journal.pbio.3004093.r003

30 Jan 2026

Attachment Submitted filename: ResponseToReviewer.docx

# Peer review: Decision Letter 2

**DOI:** 10.1371/journal.pbio.3004093.r004

**Authors:** Taylor Hart, PhD

25 Mar 2026

Dear Dr Arrenberg,

Thank you for your patience while we considered your revised manuscript "Saccadic suppression enhances saliency of ecologically relevant stimuli in the optic tectum" for publication as a Research Article at PLOS Biology. Your revised study has been evaluated by the PLOS Biology editors, the Academic Editor, and two of the original reviewers.

In light of the reviews, which you will find at the end of this email, we would like to invite you to revise the work to thoroughly address the reviewers' reports.

As you will see, while Reviewer 1 has no further concerns, Reviewer 3 highlighted several remaining issues. These points relate to the analyses, the interpretations, and unaddressed alternative explanations, as well as aspects requiring clarification. We think that some of these concerns are substantial, and the reviewer's concerns should be addressed thoroughly as part of another Major Revision.

Given the extent of revision needed, we cannot make a decision about publication until we have seen the revised manuscript and your response to the reviewers' comments. Your revised manuscript is likely to be sent for further evaluation by all or a subset of the reviewers.

In addition to these revisions, you will need to complete some formatting changes, which you will receive in a follow up email. A member of our team will be in touch with a set of requests shortly.

We expect to receive your revised manuscript within 3 months. Please email us (plosbiology@plos.org) if you have any questions or concerns, or would like to request an extension.

At this stage, your manuscript remains formally under active consideration at our journal; please notify us by email if you do not intend to submit a revision so that we may withdraw it.

**IMPORTANT - SUBMITTING YOUR REVISION**

Your revisions should address the specific points made by each reviewer. Please submit the following files along with your revised manuscript:

1. A 'Response to Reviewers' file - this should detail your responses to the editorial requests, present a point-by-point response to all of the reviewers' comments, and indicate the changes made to the manuscript.

*NOTE: In your point-by-point response to the reviewers, please provide the full context of each review. Do not selectively quote paragraphs or sentences to reply to. The entire set of reviewer comments should be present in full and each specific point should be responded to individually, point by point.

You should also cite any additional relevant literature that has been published since the original submission and mention any additional citations in your response.

2. In addition to a clean copy of the manuscript, please also upload a 'track-changes' version of your manuscript that specifies the edits made. This should be uploaded as a "Revised Article with Changes Highlighted" file type.

*Re-submission Checklist*

When you are ready to resubmit your revised manuscript, please refer to this re-submission checklist: https://plos.io/Biology_Checklist

To submit a revised version of your manuscript, please go to https://www.editorialmanager.com/pbiology/ and log in as an Author. Click the link labelled 'Submissions Needing Revision' where you will find your submission record.

Please make sure to read the following important policies and guidelines while preparing your revision:

*Published Peer Review*

Please note while forming your response, if your article is accepted, you may have the opportunity to make the peer review history publicly available. The record will include editor decision letters (with reviews) and your responses to reviewer comments. If eligible, we will contact you to opt in or out. Please see here for more details:

https://blogs.plos.org/plos/2019/05/plos-journals-now-open-for-published-peer-review/

*PLOS Data Policy*

Please note that as a condition of publication PLOS' data policy (http://journals.plos.org/plosbiology/s/data-availability) requires that you make available all data used to draw the conclusions arrived at in your manuscript. If you have not already done so, you must include any data used in your manuscript either in appropriate repositories, within the body of the manuscript, or as supporting information (N.B. this includes any numerical values that were used to generate graphs, histograms etc.). For an example see here: http://www.plosbiology.org/article/info%3Adoi%2F10.1371%2Fjournal.pbio.1001908#s5

*Blot and Gel Data Policy*

We require the original, uncropped and minimally adjusted images supporting all blot and gel results reported in an article's figures or Supporting Information files. We will require these files before a manuscript can be accepted so please prepare them now, if you have not already uploaded them. Please carefully read our guidelines for how to prepare and upload this data: https://journals.plos.org/plosbiology/s/figures#loc-blot-and-gel-reporting-requirements

*Protocols deposition*

To enhance the reproducibility of your results, we recommend that if applicable you deposit your laboratory protocols in protocols.io, where a protocol can be assigned its own identifier (DOI) such that it can be cited independently in the future. Additionally, PLOS ONE offers an option for publishing peer-reviewed Lab Protocol articles, which describe protocols hosted on protocols.io. Read more information on sharing protocols at https://plos.org/protocols?utm_medium=editorial-email&utm_source=authorletters&utm_campaign=protocols

Thank you again for your submission to our journal. We hope that our editorial process has been constructive thus far, and we welcome your feedback at any time. Please don't hesitate to contact us if you have any questions or comments.

Sincerely,

Taylor

Taylor Hart, PhD,

Associate Editor

PLOS Biology

thart@plos.org

------------------------------------

REVIEWS:

Reviewer #1: The authors adequately addressed my concerns and comments and significantly improved the manuscript!

Reviewer #3: Comparing RGC and tectal activity:

- To better compare the retinal and tectal data, the authors should do so in the same domain, rates (deconv the fluorescence), or fluorescence (convolve the rates). This is particularly problematic in Figure 8, where suppression is being compared across conditions.

- Only ~2% of the tectal cell in Figure 3 respond to flashes, while almost all of the RGC cells did. This begs the question if this tectal group is the most relevant population? What evidence is there that the suppression in these cells is behaviorally relevant, as opposed to suppression in another group?

Characterization of the suppressive effect in the tectum:

- The authors have not properly dealt with the issue that the suppressive effect in the tectum is present ahead of the time there is any texture displacement (Figure 5D). Indeed, the simplest interpretation of this figure is that the texture displacement at time 0 is not having any effect as the suppression at time -500 looks to be indistinguishable from suppression over the next 4 seconds.

What could be going on? The authors suggest there could be stimulus-stimulus interactions. The authors have the stimulus times recorded, so they should be able to demonstrate if interactions explain the issue.

Another possibility, given the results of Figure 6F, is that there are motor efference signals that are providing suppression. As seen in this figure, even in the absence of texture there is suppression that may be coupled to the time of saccade onset - hard to be definitive here since no pre-saccadic suppression is shown. If we allow that there is saccade-related suppression in the absence of texture due to efference from oculomotor control centers, then we have to expect that this would be the case even when the eyes are restrained in agarose. Thus, the pre-texture displacement suppression seen in Figure 5D could largely be due to the presence of a saccadic signal in advance of the texture displacement. This possibility would likely depend, however, on some coupling between texture displacement and the time of the following saccade.

- This lack of understanding for the pre-stimulus period also applies to Figure 1, Figure 7, and Figure 8. In turn, Figure 9 is misleading as in this cartoon the pre-stimulus suppression is always drawn as being the same as that of the baseline at time 8 seconds or later.

Clarity and writing:

- Figure 6: How many cells? What fraction of the population?

- Figure 7: Unclear how many cells are associated with this figure and if the cells under the different conditions are completely different populations.

- Many paragraphs are verbose and run-on. At times, figures are presented without reference to the results of each panel, and results are in the figure legends.

# Author comment: Author response to Decision Letter 3

**DOI:** 10.1371/journal.pbio.3004093.r005

30 May 2026

Attachment Submitted filename: Response to Reviewer_2.pdf

# Peer review: Decision Letter 3

**DOI:** 10.1371/journal.pbio.3004093.r006

**Authors:** Taylor Hart, PhD

8 Jul 2026

Dear Dr Arrenberg,

Thank you for your patience while we considered the latest revision of your manuscript "Saccadic suppression enhances saliency of ecologically relevant stimuli in the optic tectum" for consideration as a Research Article at PLOS Biology. Your revised study has now been evaluated by the PLOS Biology editors, the Academic Editor, and one of the original reviewers.

In light of the reviews, which you will find at the end of this email, we are pleased to offer you the opportunity to address the remaining points from Reviewer 3 in a revision that we anticipate should not take you very long. We will then assess your revised manuscript and your response to the reviewers' comments with our Academic Editor aiming to avoid further rounds of peer-review, although we might need to consult with the reviewers, depending on the nature of the revisions.

In your revision, you should respond to all of the reviewer's remaining points. You should discuss published behavioral data relevant to backward suppression, if performing the requested experiments is not feasible.

In addition to these revisions, you may need to complete some formatting changes, which you will receive in a follow up email. A member of our team will be in touch with a set of requests shortly. If you do not receive a separate email within a few days, please assume that checks have been completed, and no additional changes are required.

We expect to receive your revised manuscript within 1 month. Please email us (plosbiology@plos.org) if you have any questions or concerns, or would like to request an extension.

At this stage, your manuscript remains formally under active consideration at our journal; please notify us by email if you do not intend to submit a revision so that we withdraw the manuscript.

**IMPORTANT - SUBMITTING YOUR REVISION**

Your revisions should address the specific points made by each reviewer. Please submit the following files along with your revised manuscript:

1. A 'Response to Reviewers' file - this should detail your responses to the editorial requests, present a point-by-point response to all of the reviewers' comments, and indicate the changes made to the manuscript.

*NOTE: In your point-by-point response to the reviewers, please provide the full context of each review. Do not selectively quote paragraphs or sentences to reply to. The entire set of reviewer comments should be present in full and each specific point should be responded to individually.

You should also cite any additional relevant literature that has been published since the original submission and mention any additional citations in your response.

2. In addition to a clean copy of the manuscript, please also upload a 'track-changes' version of your manuscript that specifies the edits made. This should be uploaded as a "Revised Article with Changes Highlighted " file type.

*Resubmission Checklist*

When you are ready to resubmit your revised manuscript, please refer to this resubmission checklist: https://plos.io/Biology_Checklist

To submit a revised version of your manuscript, please go to https://www.editorialmanager.com/pbiology/ and log in as an Author. Click the link labelled 'Submissions Needing Revision' where you will find your submission record.

Please make sure to read the following important policies and guidelines while preparing your revision:

*Published Peer Review*

Please note while forming your response, if your article is accepted, you may have the opportunity to make the peer review history publicly available. The record will include editor decision letters (with reviews) and your responses to reviewer comments. If eligible, we will contact you to opt in or out. Please see here for more details:

https://blogs.plos.org/plos/2019/05/plos-journals-now-open-for-published-peer-review/

*PLOS Data Policy*

Please note that as a condition of publication PLOS' data policy (http://journals.plos.org/plosbiology/s/data-availability) requires that you make available all data used to draw the conclusions arrived at in your manuscript. If you have not already done so, you must include any data used in your manuscript either in appropriate repositories, within the body of the manuscript, or as supporting information (N.B. this includes any numerical values that were used to generate graphs, histograms etc.). For an example see here: http://www.plosbiology.org/article/info%3Adoi%2F10.1371%2Fjournal.pbio.1001908#s5

*Blot and Gel Data Policy*

We require the original, uncropped and minimally adjusted images supporting all blot and gel results reported in an article's figures or Supporting Information files. We will require these files before a manuscript can be accepted so please prepare them now, if you have not already uploaded them. Please carefully read our guidelines for how to prepare and upload this data: https://journals.plos.org/plosbiology/s/figures#loc-blot-and-gel-reporting-requirements

*Protocols deposition*

To enhance the reproducibility of your results, we recommend that if applicable you deposit your laboratory protocols in protocols.io, where a protocol can be assigned its own identifier (DOI) such that it can be cited independently in the future. Additionally, PLOS ONE offers an option for publishing peer-reviewed Lab Protocol articles, which describe protocols hosted on protocols.io. Read more information on sharing protocols at https://plos.org/protocols?utm_medium=editorial-email&utm_source=authorletters&utm_campaign=protocols

Thank you again for your submission to our journal. We hope that our editorial process has been constructive thus far, and we welcome your feedback at any time. Please don't hesitate to contact us if you have any questions or comments.

Sincerely,

Taylor

Taylor Hart, PhD,

Associate Editor

PLOS Biology

thart@plos.org

----------------------------------------------------------------

REVIEWS:

Reviewer #3: The authors have done a fairly thorough job responding to prior comments. A few remaining points:

Backward suppression

If as described, this is indeed quite interesting. To get a sense of how long a time-window this phenomenon covers in the zebrafish, the authors should perform behavioral experiments as done in Figure 1 and carefully determine at what delay between flash and texture displacement the effect goes away. Neural activity recordings would of course be helpful but do not appear to be necessary.

Regarding a lack of coupling between texture displacement and saccades in Figure 8B: the authors should show this data somewhere. They should also take care to avoid language that implies just because the eyes were embedded there was no motor signal.

Text in Discussion is warranted on backward suppression. In doing so, the authors should also try to explain why no backward suppression is seen in Figure S5B.

Fine-texture displacement

The effect of fine texture appears to be quite variable: it looks to be suppressive in Figure 5D, but not so in Figure S5C, and could be interpreted as enhancing in Figure 6F. This variability should be noted and discussed.

Cell numbers

The point about 2% responders and the possibility of other circuits contributing to the behavior suppression should be in the Discussion.

Legends

The legends still contain many results that should be part of the main text.

# Author comment: Author response to Decision Letter 4

**DOI:** 10.1371/journal.pbio.3004093.r007

5 Aug 2026

Attachment Submitted filename: Response to Reviewer.docx

# Peer review: Decision Letter 4

**DOI:** 10.1371/journal.pbio.3004093.r008

**Authors:** Taylor Hart, PhD

22 Sep 2026

Dear Dr Arrenberg,

Thank you for your patience while we considered your revised manuscript "Saccadic suppression enhances saliency of ecologically relevant stimuli in the optic tectum" for consideration as a Research Article at PLOS Biology. Your revised study has now been evaluated by the PLOS Biology editors, the Academic Editor and previous Reviewer 3.

As you will see, Reviewer 3 has raised two remaining points related to backward suppression and fine-texture displacement. In accordance with our previous decision and after discussing these points with the Academic Editor, we think that performing additional experiments is not critical to address these issues; however, you should still thoroughly address the reviewer's points by adding more context and caveats to the experimental descriptions and the interpretations of the results. You should also comment explicitly on the time scale difference between the reported data and those in the cited literature and discuss possible explanations, such as species differences. Please note that your next revision will be assessed editorially if at all possible.

In addition to these revisions, you may need to complete some formatting changes, which you will receive in a follow up email. A member of our team will be in touch with a set of requests shortly. If you do not receive a separate email within a few days, please assume that checks have been completed, and no additional changes are required.

We expect to receive your revised manuscript within 1 month. Please email us (plosbiology@plos.org) if you have any questions or concerns, or would like to request an extension.

At this stage, your manuscript remains formally under active consideration at our journal; please notify us by email if you do not intend to submit a revision so that we withdraw the manuscript.

**IMPORTANT - SUBMITTING YOUR REVISION**

Your revisions should address the specific points made by each reviewer. Please submit the following files along with your revised manuscript:

1. A 'Response to Reviewers' file - this should detail your responses to the editorial requests, present a point-by-point response to all of the reviewers' comments, and indicate the changes made to the manuscript.

*NOTE: In your point-by-point response to the reviewers, please provide the full context of each review. Do not selectively quote paragraphs or sentences to reply to. The entire set of reviewer comments should be present in full and each specific point should be responded to individually.

You should also cite any additional relevant literature that has been published since the original submission and mention any additional citations in your response.

2. In addition to a clean copy of the manuscript, please also upload a 'track-changes' version of your manuscript that specifies the edits made. This should be uploaded as a "Revised Article with Changes Highlighted " file type.

*Revision Checklist*

When you are ready to submit your revised manuscript, please refer to this revision checklist: https://plos.io/Biology_Checklist

To submit a revised version of your manuscript, please go to https://www.editorialmanager.com/pbiology/ and log in as an Author. Click the link labelled 'Submissions Needing Revision' where you will find your submission record.

Please make sure to read the following important policies and guidelines while preparing your revision:

*Published Peer Review*

Please note while forming your response, if your article is accepted, you may have the opportunity to make the peer review history publicly available. The record will include editor decision letters (with reviews) and your responses to reviewer comments. If eligible, we will contact you to opt in or out. Please see here for more details:

https://blogs.plos.org/plos/2019/05/plos-journals-now-open-for-published-peer-review/

*PLOS Data Policy*

Please note that as a condition of publication PLOS' data policy (http://journals.plos.org/plosbiology/s/data-availability) requires that you make available all data used to draw the conclusions arrived at in your manuscript. If you have not already done so, you must include any data used in your manuscript either in appropriate repositories, within the body of the manuscript, or as supporting information (N.B. this includes any numerical values that were used to generate graphs, histograms etc.).

*Mandatory Code Policy*

PLOS Biology requires authors to make all author-generated code publicly available without access restriction at the time of publication, unless specific legal or ethical restrictions prohibit public sharing of code, which should be disclosed (https://journals.plos.org/plosbiology/s/code-availability). Shared code needs to be made accessible for free and should be easy to locate and download. We strongly recommend that all code be deposited in a permanent, public repository that issues citable digital object identifiers (DOI) or other persistent identifiers. Where code has been shared in a non-permanent repository, such as Github or Bitbucket, we ask that a snapshot of the code is archived in a permanent, public repository, for example Zenodo, CodeOcean, or the Software Heritage archive.

*Blot and Gel Data Policy*

We require the original, uncropped and minimally adjusted images supporting all blot and gel results reported in an article's figures or Supporting Information files. We will require these files before a manuscript can be accepted so please prepare them now, if you have not already uploaded them. Please carefully read our guidelines for how to prepare and upload this data: https://journals.plos.org/plosbiology/s/figures#loc-blot-and-gel-reporting-requirements

*Protocols deposition*

To enhance the reproducibility of your results, we recommend that if applicable you deposit your laboratory protocols in protocols.io, where a protocol can be assigned its own identifier (DOI) such that it can be cited independently in the future. Additionally, PLOS ONE offers an option for publishing peer-reviewed Lab Protocol articles, which describe protocols hosted on protocols.io. Read more information on sharing protocols at https://plos.org/protocols?utm_medium=editorial-email&utm_source=authorletters&utm_campaign=protocols

Thank you again for your submission to our journal. We hope that our editorial process has been constructive thus far, and we welcome your feedback at any time. Please don't hesitate to contact us if you have any questions or comments.

Sincerely,

Taylor

Taylor Hart, PhD,

Associate Editor

PLOS Biology

thart@plos.org

----------------------------------------------------------------

REVIEWS:

Reviewer #3:

The authors have not adequately addressed some important elements of the prior review.

Backward suppression:

A concern that has been raised several times is that key data like that in Figure 5D are being presented without a proper pre-stimulus baseline. This has consistently made it difficult to causally interpret the impact of the texture displacement. The authors have tried to argue that the suppressed pre-stimulus (texture) baseline in Figure 1D and 5D is due to a form of backward masking. However, the evidence they provided in their prior rebuttal for backward masking from Idrees et al. 2022 shows an effect on the 10s of milliseconds timescale, at least an order of magnitude faster than what is being seen here. It is therefore imperative that the authors come to better grips with this issue and demonstrate the role of the texture displacement once a proper pre-stimulus (texture) is established. It will be fine to do this for the behavioral data in Figure 1D. Presumably, given the O-bend response latency, they will need to begin with flashes that start at least 2 seconds ahead of the texture displacement.

Fine-texture displacement:

Figure 6F appears to show that there is a significant suppression effect due to the eye movement alone ('No Texture') that is accentuated when the visual feedback is coarsely textured ('Coarse') and greatly reduced when the visual feedback is finely textured ('Fine'). Therefore, one would conclude from this panel that during natural behavior, some textures are actually enhancive and not suppressive relative to a no-texture condition?

# Author comment: Author response to Decision Letter 5

**DOI:** 10.1371/journal.pbio.3004093.r009

28 Sep 2026

Attachment Submitted filename: Response_to_Reviewer_auresp_5.docx

# Editor report: Decision Letter 5

**DOI:** 10.1371/journal.pbio.3004093.r010

**Authors:** Taylor Hart, PhD

2 Oct 2026

Dear Dr Arrenberg,

Thank you for your patience while we considered the latest revision of your manuscript "Saccadic suppression enhances saliency of ecologically relevant stimuli in the optic tectum" for publication as a Research Article at PLOS Biology. This revised version of your manuscript has been evaluated by the PLOS Biology editors and the Academic Editor.

Based on our Academic Editor's assessment of your revision, we are likely to accept this manuscript for publication. Please also make sure to address the following data and other policy-related requests.

IMPORTANT: Please ensure that your next revision addresses the following points:

--------------------------

**Title:

In accordance with our accessibility goals, we think that the title would benefit from adding the word 'zebrafish'. Would the following tweaked version be acceptable to you?

"Saccadic suppression enhances saliency of ecologically relevant stimuli in the zebrafish optic tectum"

**Ethics:

Please begin the Materials and Methods section with a new subheading titled "Ethics Statement". Please move all of the relevant ethics and approvals information to this subheading. Please also ensure to include any relevant approval codes or license numbers, as we didn't see any in the current version of the text.

**Data:

Thank you for uploading the data and scripts to G-node. However, we could not access them (we hit a "Not found" error for the specified URL). Please confirm that these items are accessible so that we can view them prior to formal acceptance of the paper. Please also include a DOI in the Data Availability Statement.

In addition, we have the following requirements for Data and Code Availability:

At minimum, numerical data as they underly the figures should be provided for the following figure panels:

1CD

2E

5D

6F

7D

S1A(right subpanels)

S5ABCD

Please cite the location of the data clearly in all relevant main and supplementary Figure legends, e.g. “The data underlying this Figure can be found in S1 Data” or “The data underlying this Figure can be found in https://doi.org/10.5281/zenodo.XXXXX”

Code should be documented and possess a README and a license.

**Supplement format:

Please include all of the legends for the Supplementary Figures within the main manuscript text document. THen, please upload the Supplementary Figures individually as Supporting Information files. Please also ensure that all references within the Supporting Information are included in the main references list.

--------------------------

As you address these items, please take this last chance to review your reference list to ensure that it is complete and correct. If you have cited papers that have been retracted, please include the rationale for doing so in the manuscript text, or remove these references and replace them with relevant current references. Any changes to the reference list should be mentioned in the cover letter that accompanies your revised manuscript.

In addition to these revisions, you may need to complete some formatting changes, which you will receive in a follow up email. A member of our team will be in touch with a set of requests shortly. If you do not receive a separate email within a few days, please assume that checks have been completed, and no additional changes are required.

We expect to receive your revised manuscript within two weeks.

To submit your revision, please go to https://www.editorialmanager.com/pbiology/ and log in as an Author. Click the link labelled 'Submissions Needing Revision' to find your submission record. Your revised submission must include the following:

- a cover letter that should detail your responses to any editorial requests, if applicable, and whether changes have been made to the reference list

- a Response to Reviewers file that provides a detailed response to the reviewers' comments (if applicable, if not applicable please do not delete your existing 'Response to Reviewers' file.)

- a track-changes file indicating any changes that you have made to the manuscript.

NOTE: If Supporting Information files are included with your article, note that these are not copyedited and will be published as they are submitted. Please ensure that these files are legible and of high quality (at least 300 dpi) in an easily accessible file format. For this reason, please be aware that any references listed in an SI file will not be indexed. For more information, see our Supporting Information guidelines:

https://journals.plos.org/plosbiology/s/supporting-information

*Published Peer Review History*

Please note that you may have the opportunity to make the peer review history publicly available. The record will include editor decision letters (with reviews) and your responses to reviewer comments. If eligible, we will contact you to opt in or out. Please see here for more details:

https://plos.org/published-peer-review-history/

*Press*

Should you, your institution's press office or the journal office choose to press release your paper, please ensure you have opted out of Early Article Posting on the submission form. We ask that you notify us as soon as possible if you or your institution is planning to press release the article.

*Protocols deposition*

To enhance the reproducibility of your results, we recommend that if applicable you deposit your laboratory protocols in protocols.io, where a protocol can be assigned its own identifier (DOI) such that it can be cited independently in the future. Additionally, PLOS ONE offers an option for publishing peer-reviewed Lab Protocol articles, which describe protocols hosted on protocols.io. Read more information on sharing protocols at https://plos.org/protocols?utm_medium=editorial-email&utm_source=authorletters&utm_campaign=protocols

Please do not hesitate to contact me should you have any questions.

Sincerely,

Taylor

Taylor Hart, PhD,

Associate Editor

thart@plos.org

PLOS Biology

Comments from the Academic Editor:

I am satisfied with the authors' responses to the concerns of Reviewer 3. I recommend accepting the manuscript.

# Author comment: Author response to Decision Letter 6

**DOI:** 10.1371/journal.pbio.3004093.r011

8 Oct 2026

Attachment Submitted filename: Response_to_Reviewer_auresp_6.docx

# Editor report: Decision Letter 6

**DOI:** 10.1371/journal.pbio.3004093.r012

**Authors:** Taylor Hart, PhD

19 Oct 2026

Dear Dr Arrenberg,

Thank you for the submission of your revised Research Article "Saccadic suppression enhances saliency of ecologically relevant stimuli in the zebrafish optic tectum" for publication in PLOS Biology. On behalf of my colleagues and the Academic Editor, Jonathan Demb, I am pleased to say that we can in principle accept your manuscript for publication, provided you address any remaining formatting and reporting issues. These will be detailed in an email you should receive within 2-3 business days from our colleagues in the journal operations team; no action is required from you until then. Please note that we will not be able to formally accept your manuscript and schedule it for publication until you have completed any requested changes.

Please take a minute to log into Editorial Manager at http://www.editorialmanager.com/pbiology/, click the "Update My Information" link at the top of the page, and update your user information to ensure an efficient production process.

PRESS

We frequently collaborate with press offices. If your institution or institutions have a press office, please notify them about your upcoming paper at this point, to enable them to help maximise its impact. If the press office is planning to promote your findings, we would be grateful if they could coordinate with biologypress@plos.org. If you have previously opted in to the early version process, we ask that you notify us immediately of any press plans so that we may opt out on your behalf.

We also ask that you take this opportunity to read our Embargo Policy regarding the discussion, promotion and media coverage of work that is yet to be published by PLOS. As your manuscript is not yet published, it is bound by the conditions of our Embargo Policy. Please be aware that this policy is in place both to ensure that any press coverage of your article is fully substantiated and to provide a direct link between such coverage and the published work. For full details of our Embargo Policy, please visit http://www.plos.org/about/media-inquiries/embargo-policy/.

Thank you again for choosing PLOS Biology for publication and supporting Open Access publishing. We look forward to publishing your study.

Sincerely,

Taylor

Taylor Hart, PhD,

Associate Editor

PLOS Biology

thart@plos.org
